# Supplementary material for: Novel Nucleic Acid Detection for Human Parvovirus B19 Based on Pyrococcus furiosus Argonaute Protein
Source: Viruses. 2023 Feb 21;15(3):595. doi: 10.3390/v15030595 (PMC10057799; doi:10.3390/v15030595)
Supplement: Supplementary file 1 [file viruses-15-00595-s001.zip › viruses-211868-supplementary.pdf]

viruses

Supplementary Data

Supplementary Materials and Methods

Oligos, enzymes, and reagents

The primers, gDNA, ssDNA(MB-B19) and B19V tDNA(B19 tDNA) were synthesized by Sangon Biotech (Shanghai, China). Moreover, all the nucleic acid sequences are summarized in Supplementary Table 1. T4 polynucleotide kinases(PNK) were purchased from New England Biolabs (MA, United States). 2×HieffTM PCR Master Mix obtained from Yeason Biotech(Shanghai, China) was used to measure the PCR reaction assays.The High Pure viral DNA Extraction Kit purchased from Megan (Wuhan, China) was validated for the viral DNA extraction.

PCR and nested PCR assay

For amplification of B19V NS1 PCR product target DNA used in PAND detection, the primers NS1-F/R was used for PCR assays, the primers NS-2-F/R(first round) and NS1-F/R(second round) was used for nested PCR assays. Each 10 μL PCR reaction mixture contained 3pmol each primer, 5 μL 2×HieffTM PCR Master Mix, and 50ng DNA or 2μL Clinical sample DNA. Cycling conditions were: 95°C for 3min; then 35 cycles of 94°C for 20s, 58°C for 10s and 72°C for 10 s; followed by a final extension of 72°C for 5min. For nested PCR, the first and second round of PCR program consisted of initial denaturation at 95°C for 3 min; then 30 cycles of 94°C for 20s, 58°C for 10s and 72°C for 10 s; followed by a final extension of 72°C for 5min. 1μL of 10-fold-diluted first-round PCR products as template was used for second round of PCR.

Table S1. Oligonucleotides and primers used in this report.

Name	Sequence（5' to 3'）
Positive target	AAACGACGGCCAGTGCCAAGCTTACTATACAACCTACTACCTCAT
gDNA	TGAGGTAGTAGGTTGTAT
gr-B19	AGTTAACCATGCCATA
gt-B19	TTGCTAAAAGTGTTCC
gf-B19	AGTATATGGCATGGTT
gn	GCCATATACTGGAACA
gn-2	GCCATATACTGGAACACTTTTAGCAATGGCCATTGCCAAGTTTGTTTTTCC
MB-B19	CGCACCTGTTCCAGTATATGGCGGTGCG
NS1-2-F1	TATTGGTGGGGCAGCATGTGTTAAAGT
NS1-2-R1	AGCTGCTTTCACTGAGTTCTTCAGAG
NS1-F	AATACACTGTGGTTTTATGGGCCG
NS1-R	ACCATTGCTGGTTATAACCACAGGT
B19-tDNA-F	ATGGCCATTGCTAAAAGTGTTCCAGTATATGGCATGGTTAACTGGA
B19-tDNA-R	TCCAGTTAACCATGCCATATACTGGAACACTTTTAGCAATGGCCAT

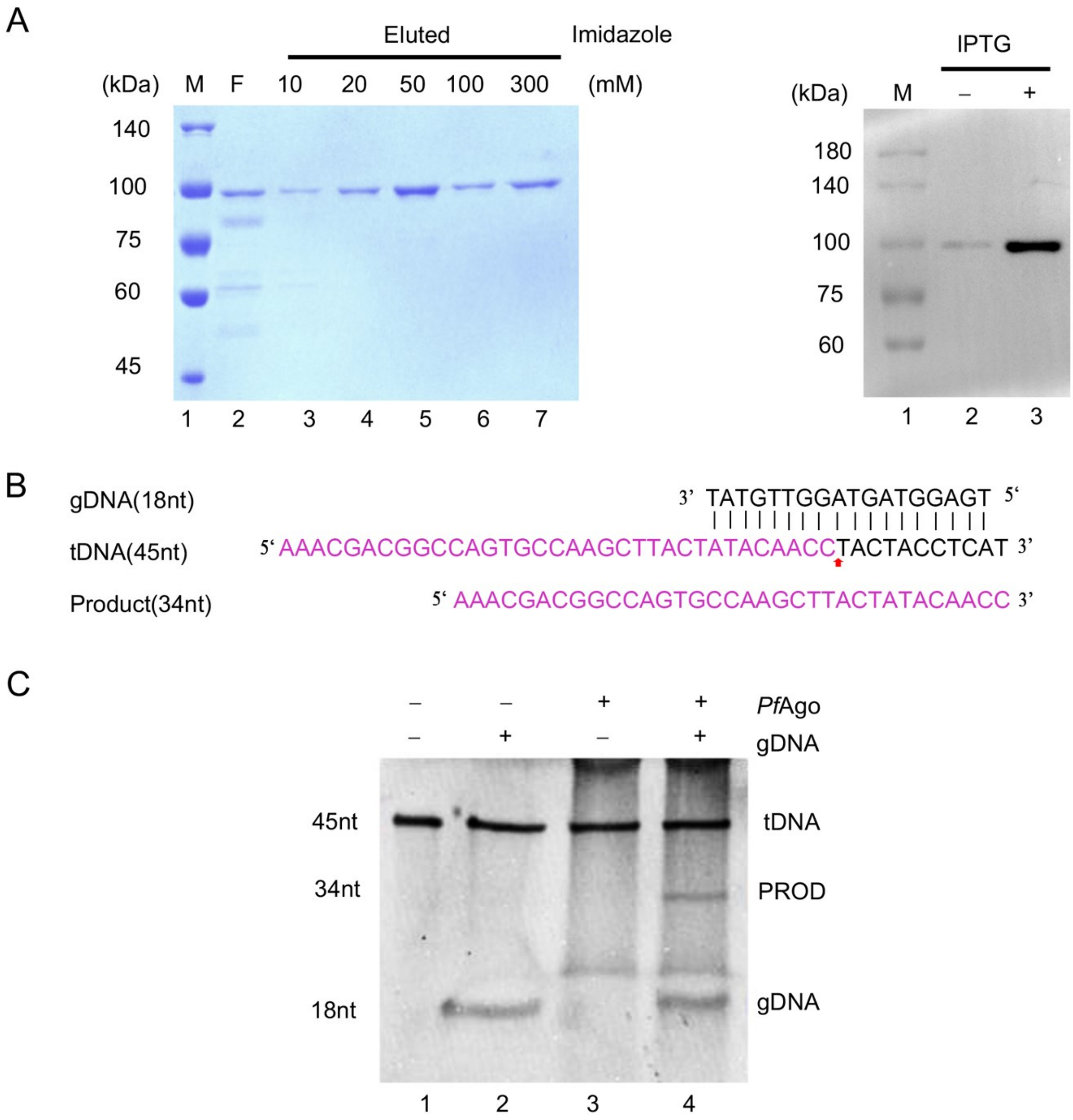

Figure S1. Endonuclease activity assay of purified His-*Pf*Ago. A. SDS-PAGE (left) and Western blot(right) assay of the purified His-*Pf*Ago fusion protein. M: Protein ladder, F: Flowthrough. **B**. Schematic of the standard reaction assay showed the target ssDNA(45nt), the phosphorylated gDNA(5'P-gDNA), and the cleavage sites. The cleavage band was indicated in red. **C**. Identification of standard endonuclease cleavage reaction. His-*Pf*Ago protein was loaded with an 18nt gDNA and was incubated with a 45 nt ssDNA target at a 90:4:1 molar ratio (*Pf*Ago: guide: target) as described in Materials and Methods. Next, the cleaved products were analyzed on a 20% denaturing polyacrylamide gel. The sizes of the target, gDNA, and cleavage product bands are shown on the left. tDNA: target ssDNA, CP: Cleavage products, gDNA: guide DNA, nt: nucleotide. And the control sample contains no His-*Pf*Ago protein.

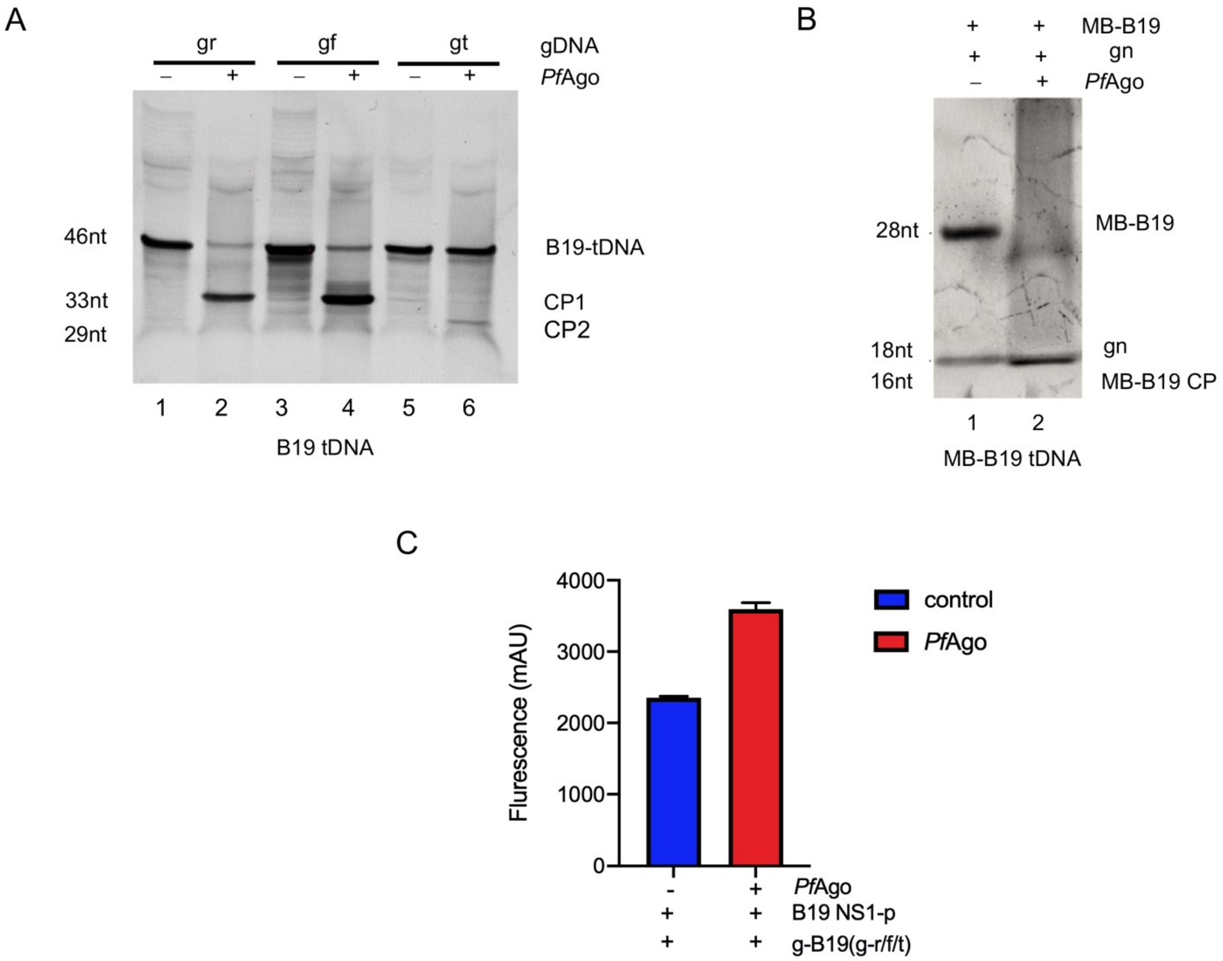

Figure S2. Establishment of the *Pf*Ago-mediated B19V nucleic acid detection system. A. B19V ssDNA target cleavage efficient assay based on phosphorated gr/gf-/gt was shown on 20% TBE-PAGE electrophoresis. The sizes of the target, gDNA, and cleavage product bands are indicated on the left. gr: 5'P-gr-B19, gr: 5'P-gf-B19, gt: 5'P-gt-B19, CP1: Cleavage product mediated with gr and gf, CP2: Cleavage product mediated with gt, B19 tDNA: B19V target ssDNA. As described in the text, **B**. gn mediated *Pf*Ago-specific cleavage of MB-B19 was indicated on 20% TBE-PAGE electrophoresis. The size of gn(16nt) and the cleavage band MB-B19 CP(16nt) was shown on the left. **C**. Fluorescence intensity detection of B19-NS1 PAND based on three phosphorated gr/gf/gt as mentioned in Figure1B was indicated. Error bars represent mean ± SD, where n = 3 replicates.

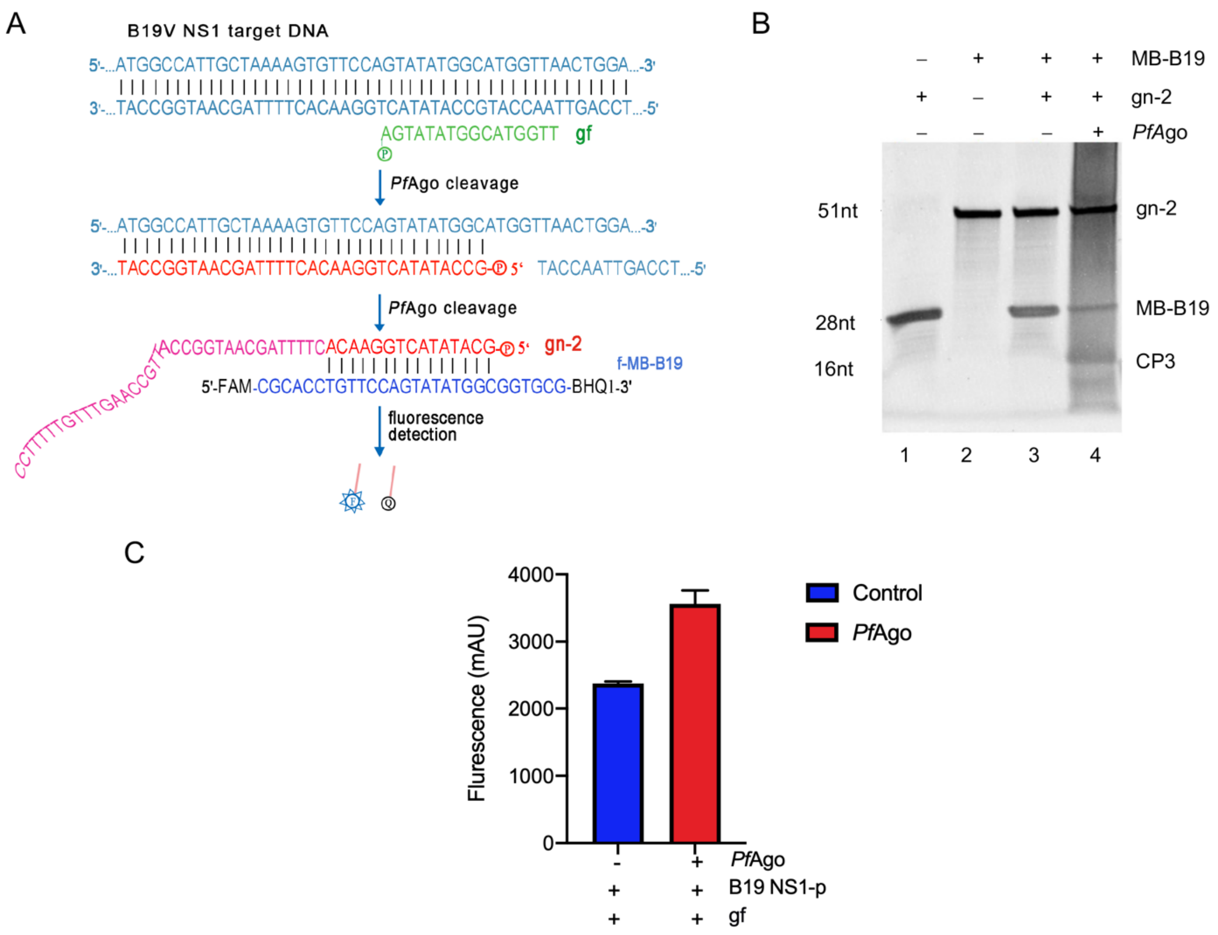

Figure S3. Establishment of the B19-NS1 PAND with one single gDNA system. A. Schematic of B19-NS1 PAND mediated with one single gDNA. As described in the text, **B**. g-MB-B19-2 mediated *Pf*Ago-specific cleavage of MB-B19 was indicated on 20% TBE-PAGE electrophoresis. **C**. Fluorescence intensity detection of B19-NS1 PAND based on phosphorated gf as mentioned in Figure1C, was indicated. Error bars represent mean ± SD, where n = 3 replicates.

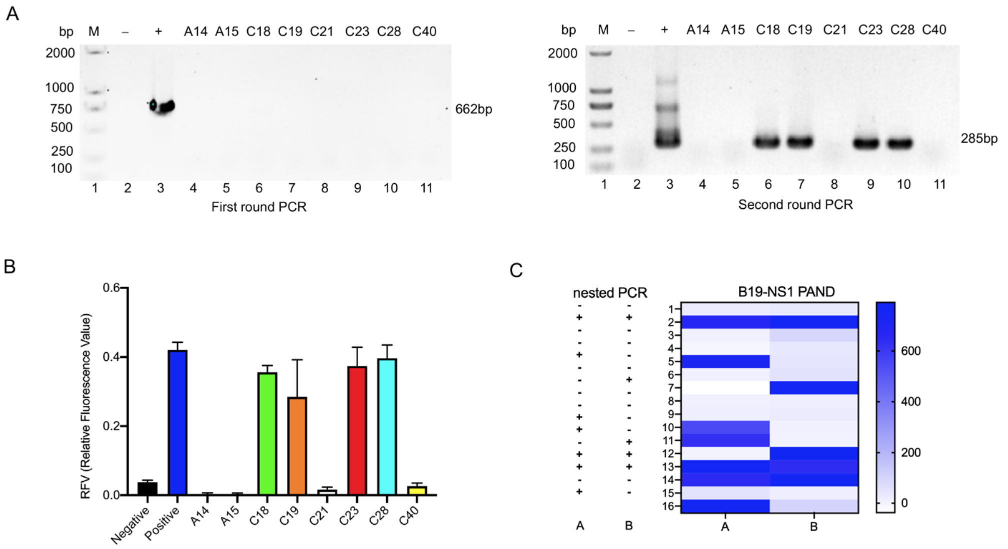

Figure S4. Analysis of clinical samples by B19-NS1 PAND coupled with nested PCR. Eight clinical samples (A14, A15, C18, C19, C21, C23, C28, C40) measured by nested PCR assay was indicated on agarose gel electrophoresis with Ethidium bromide staining **(A)** or by three

guides-mediated B19-NS1 PAND coupled with nested PCR was detect and shown **(B)**. (**C)** 32 clinical samples measured by B19-NS1 PAND preamplification with nested PCR show 100% concordance with nested PCR assay based on agarose gel electrophoresis.
